# The expression of autophagy markers in IVIG-resistant Kawasaki disease and the establishment of prediction model

**DOI:** 10.1186/s12887-023-04386-3

**Published:** 2023-12-19

**Authors:** Yang Zhou, Yali Wu, Chunhui Yuan, Wei Yin, Baoxiang Wang, Yan Ding

**Affiliations:** 1 Department of Immunology and Infectious Diseases, Wuhan Children’s Hospital, Tongji Medical College, Huazhong University of Science and Technology, Wuhan, 430016 China; 2 Department of Laboratory Medicine, Wuhan Children’s Hospital, Tongji Medical College, Huazhong University of Science and Technology, Wuhan, 430016 China; 3 Department of Digestive System, Wuhan Children’s Hospital, Tongji Medical College, Huazhong University of Science and Technology, Wuhan, 430016 China

**Keywords:** Autophagy markers, Kawasaki disease, Immunoglobulin-resistant, Predictors, Independent risk factors, Scoring model

## Abstract

**Background:**

The aim of this study was to find early predictors of Intravenous Immunoglobulin (IVIG)-Resistant Kawasaki Disease.

**Methods:**

Patients diagnosed with Kawasaki disease were enrolled in this study. Univariate analysis and multiple logistic regression were used to analyze the clinical characteristics and laboratory findings of patients in both groups before IVIG treatment. Independent predictors of Intravenous Immunoglobulin-Resistant Kawasaki Disease were analyzed, and a prediction model for children with Intravenous Immunoglobulin-Resistant Kawasaki Disease was constructed.

**Results:**

A total of 108 children (67 males and 41 females) with IVIG-sensitive Kawasaki disease and 31 children (20 males and 11 females) with IVIG-resistant Kawasaki disease participated in this study. Compared with the IVIG-sensitive group, the duration of hospitalization, ALT, AST, GLB, r-GT, IgG, PCT, and ESR was elevated in the IVIG-resistant KD group, and ATG16L1, LC3II, BECN1, RBC, HGB, ALB, A/G, and CK were significantly lower (*P* < 0.05). mRNA expression of ESR, BECN1, and LC3II were independent risk factors for IVIG-resistant Kawasaki disease. A logistic regression model and scoring system were established, and the cut-off values of independent risk factors were derived from ROC curves: ESR ≥ 79.5 mm/h, BECN1 ≤ 0.645, LC3II ≤ 0.481. A new scoring system was established according to the respective regression coefficients as follows: ESR ≥ 79.5 mm/h (1 point), BECN1 ≤ 0.645 (1 point). LC3II ≤ 0.481 (2 points), 0–1 as low risk for IVIG non-response, and ≥ 2 as high risk. Applied to this group of study subjects, the sensitivity was 87.10%, specificity 83.33%, Youden index 0.70, AUC 0.9.

**Conclusions:**

Autophagy markers ATG16L1, BECN1, and LC3II are down-regulated in the expression of IVIG -resistant KD. ESR, BECN1, and LC3II mRNAs are independent risk factors for IVIG-resistant KD and may be involved in the development of IVIG-resistant KD. This study established a new model that can be used to predict IVIG-resistant KD, and future validation in a larger population is needed.

## Introduction

In 1967, the Japanese scholar Tomisaku Kawasaki reported a case of fever, rash, and strawberry tongue, hence the name Kawasaki disease (KD) [1]. Epidemiology shows geographical and seasonal differences in the incidence of KD, with the highest incidence in East Asia and an increase in the incidence of Kawasaki disease over the past few years [2–6]. Approximately 25% of children with untreated KD develop coronary artery lesion (CAL)) [7], which has been the most frequent cause of acquired heart disease in developed countries [8]. The current treatment of choice is oral aspirin combined with intravenous immunoglobulin (IVIG) 2 g/kg [7], but still about 10%-20% of children who do not respond to IVIG therapy or reappear clinically about 2 days after the end of treatment are more likely to develop CAL [9, 10]. The IVIG resistant KD was defined as “recrudescent or persistent fever at least 36 h following completion of the first dose of IVIG.” by The AHA [7], But some studies used 24 h or 48 h instead of 36 h.

The early identification of IVIG resistant KD is particularly important, so many experts at home and abroad have established many scoring models for this purpose, mostly based on the age of the child, clinical characteristics, time of first IVIG use, etc. The main early scoring criteria for IVIG resistant KD are the Egami score, Kobayashi score, Sano score, Fu score, etc. In 2006 Kobayashi used seven variables such as age, time of initial IVIG use, CRP, neutrophil ratio, platelet count, serum sodium, and aspartate aminotransferase as predictors of IVIG resistant KD when more than three of these variables were abnormal for IVIG resistant KD [11]. However, the specificity and sensitivity of this model as well as other scoring methods in clinical validation were < 75%. Yang established a Chinese scoring criterion in 2019: TB > 20 umol/L (5 points), CRP ≥ 90 mg/L and serum sodium < 135 mmol/L were each recorded as 3 points; percentage of neutrophils ≥ 70% and albumin < 35 g/L were each recorded as 2.5 points; a total of 16 points, and when the score ≥ 6 was classified as a high-risk group, the model had a sensitivity of only 56% and specificity of 79% [12]. In clinical practice, these predictors can be obtained through blood tests. However, to date there is no scoring system that is applicable to geographic and ethnic differences worldwide.

Autophagy is a general term for the pathway by which cytoplasmic material is delivered to lysosomes for degradation and includes types such as macroautophagy, chaperone-mediated autophagy (CMA), and microautophagy [13]. Autophagy has the ability to regulate the secretion of cytokines from immune cells [14]. The interaction between autophagy and inflammation may be manifested by the role of autophagy on the induction of inflammatory vesicles and IL-1β secretion. Autophagy can also regulate the secretion of cytokines such as IL-6, IL-18 and TNF-α [15]. There is growing evidence that autophagy can restore cellular function and reduce the pro-inflammatory state under pathological conditions thereby reducing vascular inflammation in the heart [16]. In a mouse model of Kawasaki disease enhanced autophagic flux significantly reduced cardiovascular lesions in mice, and blocking autophagy increased inflammation [17]. Huang et al. demonstrated that mRNA levels of autophagic markers LC3II, BECLIN1 and ATG16L1 were downregulated in leukocytes of children with KD but significantly increased after immunoglobulin treatment, and in the coronary injury group ATG16L1 expression was consistently down-regulated [18]. The aim of this study was to collect and analyze clinical data and expression of autophagy markers in children with Kawasaki disease, to identify independent risk factors and to develop a valuable predictive model for IVIG resistant KD.

## Materials and methods

### Patients

**Fig. 1:**
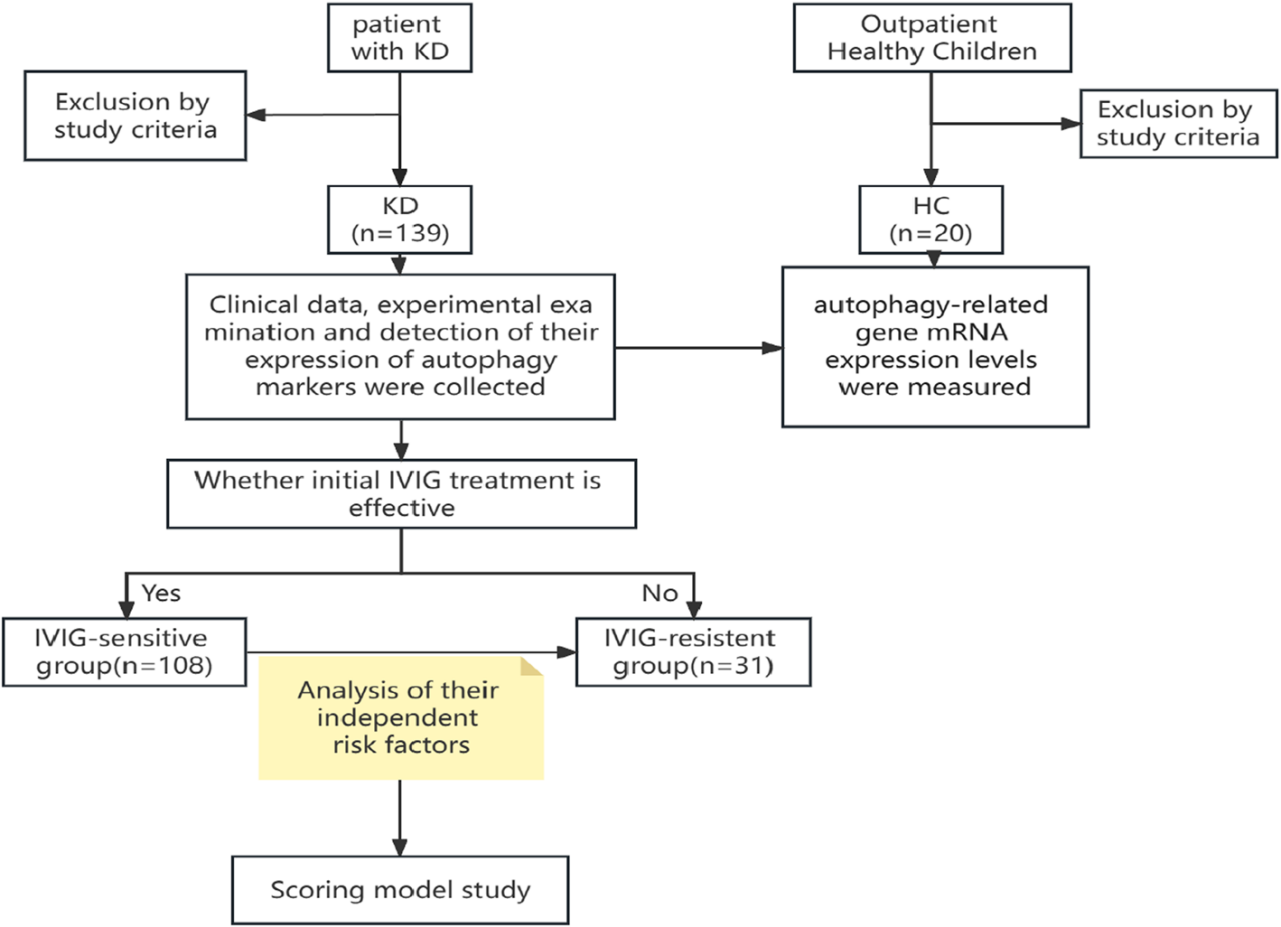
Flow chart of study subject inclusion

This study was approved by the Accreditation Committee of Wuhan Children's Hospital (No.2021R074-E02), and informed consent was obtained from the children's parents or guardians. All children diagnosed with KD were treated with IVIG (2 mg/kg) by slow infusion. At the onset (prior to IVIG treatment; KD1). Patients who did not meet the diagnostic criteria for IVIG-Resistant KD were excluded. Thirty-one children with IVIG resistant KD were included in this study, and 108 children with IVIG-sensitive Kawasaki disease were used as controls. From the outpatient clinic, we recruited 20 additional healthy children (without any history of KD) and volunteered to participate in our study as healthy controls (Fig. 1). The diagnosis of KD was referred to the 2017 American Heart Association (AHA) joint guidelines for the diagnosis and treatment of KD [7].

### Criteria for the diagnosis of classic KD

The patient must have ≥ 5 days of fever as well as ≥ 4 of the 5 principal clinical features. Five of these clinical characteristics were as below: (1) conjunctival congestion; (2) Mucosal changes: red lips, strawberry tongue; (3) Polymorphous rash; (4) Extremity changes: redness and swelling of palms and feet in the initial phase, and peeling of skin at the ends of limbs in the recovery phase; and (5) non-suppurative cervical lymphadenitis [7].

Criteria for diagnosis of IVIG-resistant KD: recrudescent or persistent fever at least 36 h following completion of the first dose of IVIG [7], But some studies used 24 h or 48 h instead of 36 h [19].

### Inclusion criteria

(1) initial treatment regimen of IVIG2g/kg; (2) body temperature > 38.5 °C after 48 h of initial IVIG; (3) those taking aspirin 30-50 mg/kg*d standard dose; (4) complete clinical information required.

### Exclusion criteria

(1) incomplete KD; (2) IVIG use prior to admission; (3) glucocorticoid use prior to admission;(4) Failure to use a single IVIG2g/kg infusion regimen; (5) Combination of other immunodeficiencies or diseases that affect disease progression; (6) The required clinical information is lacking.

#### Data collection

Collection of Kawasaki disease case information from hospital case systems:

1. General information and hospitalization status, including gender, age, height, weight, number of days in the hospital.
2. Clinical symptoms: duration of fever before admission, swelling of hands and feet, polymorphic rash, enlarged lymph nodes in the neck, conjunctival congestion, prune tongue, perianal desquamation, CAL.
3. Laboratory indicators: a) Routine blood: white blood cell count (WBC), neutrophil percentage (N%), platelet count (PLT), hemoglobin (HB), red blood cell count (RBC), eosinophil percentage (EOS%), monocyte percentage (MO%); b):Blood biochemistry: blood sodium (NA), blood potassium (K) total protein (TP), prealbumin (PA), total bilirubin (TB), direct bilirubin (DBIL), albumin (ALB), white globule ratio (A/G), glutamate aminotransferase (AST), r-glutamyl transpeptidase (r-GT), glutamate aminotransferase (ALT), lactate dehydrogenase (LDH), globulin (GLB), lactate dehydrogenase isozyme-1 (LDH-1), creatine phosphate kinase (CK)), alkaline phosphatase (ALP), phosphocreatine kinase isoenzyme (CK-MB); c):Immune function: percentage of CD3 + CD8 + T lymphocytes, immunoglobulin A (IgA), complement C3c, complement C4, CD3 + T lymphocytes, percentage of natural killer cells, percentage of CD3 + CD4 + T lymphocytes, immunoglobulin M (IgM), immunoglobulin G (IgG), CD3 + CD4 + CD8 + T-lymphocyte count, CD3 + CD4 + CD8 + T-lymphocyte percentage, natural killer cell count(NK), CD3 + CD8 + T-lymphocyte count, CD19 + B-lymph percentage; inflammatory indexes: sedimentation (ESR), procalcitonin (PCT), interleukin-2 (IL-2), interleukin-4 ( IL-4), interleukin-6 (IL-6), interleukin-10 (IL-10), tumor necrosis factor-α (TNF-α), C-reactive protein (CRP), ferritin (SF).

### Experimental method

Real-time fluorescence quantitative PCR to detect the expression of autophagic standards.

We collected 2 mL venous blood samples from all participants and extracted total RNA from leukocytes of KD patients and healthy controls. reverse transcription step was used) in a 20 μL reaction for 5 μl RNA, 4ul 4gwiper mix,5xHiscript surper MIX 4ul,7ul RNase Free ddH _2_O. Total volumes were performed at 20 μL per tube Reverse transcription was performed in a PCR instrument programmed to run reverse transcription at 50 °C for 15 min and inactivate reverse transcriptase at 85 °C for 5 s, and finally incubate the cDNA product at 4 °C. Prior to the PCR step, the cDNA product is stored at -20 °C.

Perform real-time fluorescent quantitative PCR experiments with the ABI 7500 Rapid Real-Time Fluorescent PCR System (Applied Biosystems). The reagents were prepared using Rapid SYBR Green Premix and following the manufacturer's instructions. The reaction mixture contains 5 μL of sample with 15 μL of PCR. each well was performed as follows: 20 s reaction at 95 °C, followed by 3 s at 95 °C and 30 s at 60 °C and repeated for 40 cycles. The dissociation phase was carried out as follows: 95 °C for 15 s, 60 °C for 60 s, 95 °C for 15 s and 60 °C for 15 s. ABI7500 software was used to obtain the raw fluorescence data for analysis using the comparative threshold cycle (2.^ΔΔCt^) equation with endogenous *GAPDH* expression as an internal control. The primers used for amplification were listed in Table 1.

**Table 1:** Primer sequences used for real time RT-PCR

Gene	Primer sequence (5’ → 3’)
ATG16L1	F: 5'- AACGCTGTGCAGTTCAGTCC-3'
	R: 5'- AGCTGCTAAGAGGTAAGATCCA-3'
BECN1	F:5'- CCATGCAGGTGAGCTTCGT-3'
	R:5'- GAATCTGCGAGAGACACCATC -3'
P62	F: 5'- GCACCCCAATGTGATCTGC-3'
	R:5'- CGCTACACAAGTCGTAGTCTGG-3'
LAMP2	F: 5'-GAAAATGCCACTTGCCTTTATGC-3'
	R: 5'-AGGAAAAGCCAGGTCCGAAC-3'
LC3II	F:5'-GATGTCCGACTTATTCGAGAGC-3'
	R: 5'-TTGAGCTGTAAGCGCCTTCTA-3'
GADPH	F:5'-GGTGAAGGTCGGAGTCAACGG-3'
	R:5'-GGTCATGAGTCCTTCCACGATCATACC-3'

#### Statistical analysis

This study used SPSS 25.0 for data analysis. Data for continuous variables conforming to a normal distribution were compared for differences by t-test, otherwise they were compared by Mann–Whitney U-test. Differences in dichotomous variables were compared by chi-square test. Variables that were statistically different between groups were first subjected to one-way logistic analysis (*P* < 0.05) to determine whether each indicator had an effect on IVIG nonresponse and to obtain OR. using a multi-factor logistic model, indicators that had value in the one-way logistic regression were included in the model together, and those that had value in the regression were screened and their corrected ORs were obtained. Finally, a predictive scoring model was developed by scoring the ratio (OR) of each independent risk factor and aggregating the total score of each child's score. The maximum Youden index corresponding to the total score threshold, sensitivity and specificity was calculated using the subject operating characteristic (ROC) curve. Statistical differences were set at *P* < 0.05.

## Results

A total of 108 children (67 males and 41 females) with IVIG-sensitive Kawasaki disease and 31 children (20 males and 11 females) with IVIG-resistant Kawasaki disease were included in the study. There were no significant differences in age, sex, height, and weight among the children in each group (*P* > 0.05, Table 2).

**Table 2:** Demographic features between IVIG-sensitive and IVIG-resistant KD children

Demographic features	IVIG-sensitive (*n* = 108)	IVIG-resistant (*n* = 31)	Z/χ2	*P*
Gender (M/F)	67/41	20/11	0.063	0.801
Age (year)	3.01 (1.64,4.35)	2.8 (1.82,3.86)	-0.941	0.347
Height (cm)	96 (83.25,110)	92 (83,104)	-0.681	0.496
Weight (kg)	14 (11,17.88)	14 (11.5,16)	-0.388	0.698

### Comparison of laboratory parameters between the IVIG-sensitive group and the IVIG-resistant group

Compared with the IVIG-sensitive group, the duration of hospitalization, ALT, AST, GLB, r-GT, IgG, PCT, and ESR were elevated and RBC, HGB, ALB, A/G, and CK were significantly lower in the IVIG-resistant Kawasaki disease group (*P* < 0.05). Pre-admission fever duration, hand and foot swelling, polymorphic rash, enlarged cervical lymph nodes, conjunctival congestion, prune tongue, perianal desquamation, CAL, WBC, N%, PLT, EOS%, MO%, NA, K, TP, PA, TB, DBIL, AST, ALT, ALP, LDH, LDH-1, CK-MB, IgA, IgM, complement C3c, complement C4, CD3 + T lymphocytes, CD3 + CD4 + CD8 + T lymphocyte count, NK, CD3 + CD8 + T lymphocyte count, CD3 + CD4 + CD8 + T lymphocyte percentage, CD3 + CD8 + T lymphocyte percentage, CD19 + B lymph percentage, natural killer cell percentage, CD3 + CD4 + T lymphocyte percentage, CRP, SF, IL-2, IL-4, IL-6, IL-10, and TNF-α did not change significantly (*P* > 0.05, Tables 3 and 4).

**Table 3:** clinical manifestations between IVIG-sensitive and IVIG-resistant KD children

Variable(s)	IVIG-sensitive(*n* = 108)	IVIG-resistant (*n* = 31)	Z/χ2	*P*
Fever before IVIG	5(5,7)	5(4,6)	-1.872	0.061
Length of hospitalization	5(4,6)	7(6,9)	4.934	< 0.001*
Stiffness of hands	73/108	21/31	0	0.998
Rash	36/108	11/31	0.05	0.823
Enlarged lymph nodes	24/108	11/31	2.249	0.134
Conjunctival congestion	95/108	28/31	0.132	0.717
Strawberry tongue	96/108	29/31	0.577	0.447
Perianal peeling	56/108	22/31	3.574	0.059
CAL	9/108	7/31	3.503	0.061

^*^P < 0.05

**Table 4:** Blood indicators between IVIG-sensitive and IVIG-resistant KD children

Variable(s)	IVIG-sensitive (*n* = 108)	IVIG-resistant (*n* = 31)	Z	*P*
WBC(× 10^9/L)	10.96(8.17,15.57)	13.38(8.09,15.79)	0.806	0.42
HB(g/L)	109(100,117)	100(90,114)	-2.787	0.005*
PLT(× 10^9/L)	358(269,439)	296(267,440)	-0.548	0.583
RBC(× 10^9/L)	3.98(3.75,4.33)	3.77(3.54,3.98)	-3.112	0.002*
N%	66.8(50.4,78.8)	66.7(52.4,82.6)	0.536	0.592
L%	23.8(14.5,36.6)	25.2(11.3,36.5)	-0.439	0.661
MO%	5.4(3.9,7.5)	6.3(3.3,8)	-0.018	0.986
EOS%	0.18(0.06,0.43)	0.12(0.03,0.35)	-1.094	0.274
ALT (U/L)	18(9.25,40.5)	24(15,108)	2.04	0.041*
AST (U/L)	26(20,40)	48(29,63)	3.394	0.001*
PA (mg/L)	82.1(51.28,111)	65.8(34.8,108.8)	-1.045	0.296
ALB (g/L)	38.95(34.5,41.58)	34.2(32.3,38.6)	-3.36	0.001*
GLB (g/L)	23.1(19.73,25.93)	24.4(20.6,38.9)	2.168	0.03*
A/G	1.7(1.4,2)	1.4(0.8,1.8)	-3.232	0.001*
TP (g/L)	61.75(57.25,66.9)	59.8(56.2,70)	0	1
r-GT(U/L)	14.5(9,59)	38(19,100)	2.592	0.01*
DBIL (μ mol/L)	3.05(2.2,4.38)	2.7(2,21.2)	0.245	0.806
TB (μmol/L)	7.85(4.83,10.15)	6.6(3.7,31.2)	-0.104	0.917
ALP(U/L)	162(129.25,207.75)	179(135,227)	1.151	0.25
NA (mmol/L)	136.05(134,138.53)	134.85(133.8,137.13)	-1.135	0.189
CK(U/L)	51(32.25,88.5)	31(25,53)	-2.69	0.007*
CK-MB (U/L)	24(17,35.5)	22(17,34)	0.514	0.607
LDH(U/L)	285.5(239.5,384.5)	290(224,414)	-0.243	0.808
LDH-1 (U/L)	53(46,70)	57(43,76)	0.581	0.561
K(mmol/L)	4.45(3.8,4.8)	3.99(3.71,4.99)	-0.867	0.386
IgA(g/L)	0.79(0.52,1.34)	0.97(0.54,1.35)	0.663	0.507
IgM(g/L)	0.99(0.75,1.24)	0.95(0.8,1.57)	0.66	0.509
IgG(g/L)	7.46(5.84,10.08)	8.07(7.18,20)	2.378	0.017*
C3(g/L)	1.26(1.03,1.44)	1.24(1.11,1.53)	1.05	0.294
C4(g/L)	0.27(0.21,0.34)	0.24(0.2,0.28)	-1.485	0.138
CD3 + T(/μL)	1475(727.75,2230.75)	1321(720.5,3213)	0.743	0.457
CD3 + CD4 + CD8 + T (/μ L)	3(1,7)	4(1,10.5)	1.31	0.19
NK (/μ L)	162(85.25,323)	133.5(69.25,307.5)	-0.83	0.407
CD3 + CD8 + T (/μ L)	435.5(270.25,743)	534(228,981.5)	0.726	0.468
CD19 + B%	48.81(28.97,773)	70.48(34.26,661)	0.681	0.496
CD3 + CD8 + T%	18.29(13.82,22.5)	16.79(14.86,22.75)	0.334	0.738
CD3 + CD4 + T%	32.12(26.71,40.32)	37.34(25.37,42.9)	1.103	0.27
CD3 + CD4 + CD8 + T%	0.12(0.04,0.22)	0.16(0.07,0.26)	1.304	0.192
NK%	6.99(4.06,11.07)	6.15(3.92,9.19)	-1.097	0.273
ESR (mm/h)	51(20,78)	83.5(24,91)	2.826	0.005*
CRP (mg/L)	85.4(45.1,125)	75.9(33.98,126.25)	0.812	0.417
SF (ng/ml)	161.78(125.11,240.02)	175.47(123.9,323.64)	1.232	0.218
PCT (ng/mL)	0.92(0.36,2.57)	1.22(0.43,8.74)	2.252	0.024*
IL-2(pg/ml)	2.44(1.35,3.65)	3.04(1.4,3.91)	1.193	0.233
IL-4(pg/ml)	2.63(1.65,3.43)	3.1(1.82,3.93)	1.75	0.08
IL-6(pg/ml)	58.79 (21.72,145.6)	211.39(87.32,294.19)	1.827	0.068
IL-10(pg/ml)	10.53(5.98,25.31)	15.58(5.45,57.76)	0.315	0.753
TNF-α(pg/ml)	3.52(2.39,5)	3.64(2.44,5.7)	0.706	0.48

^*^P < 0.05

### Comparison of expression of autophagy markers

**Fig. 2:**
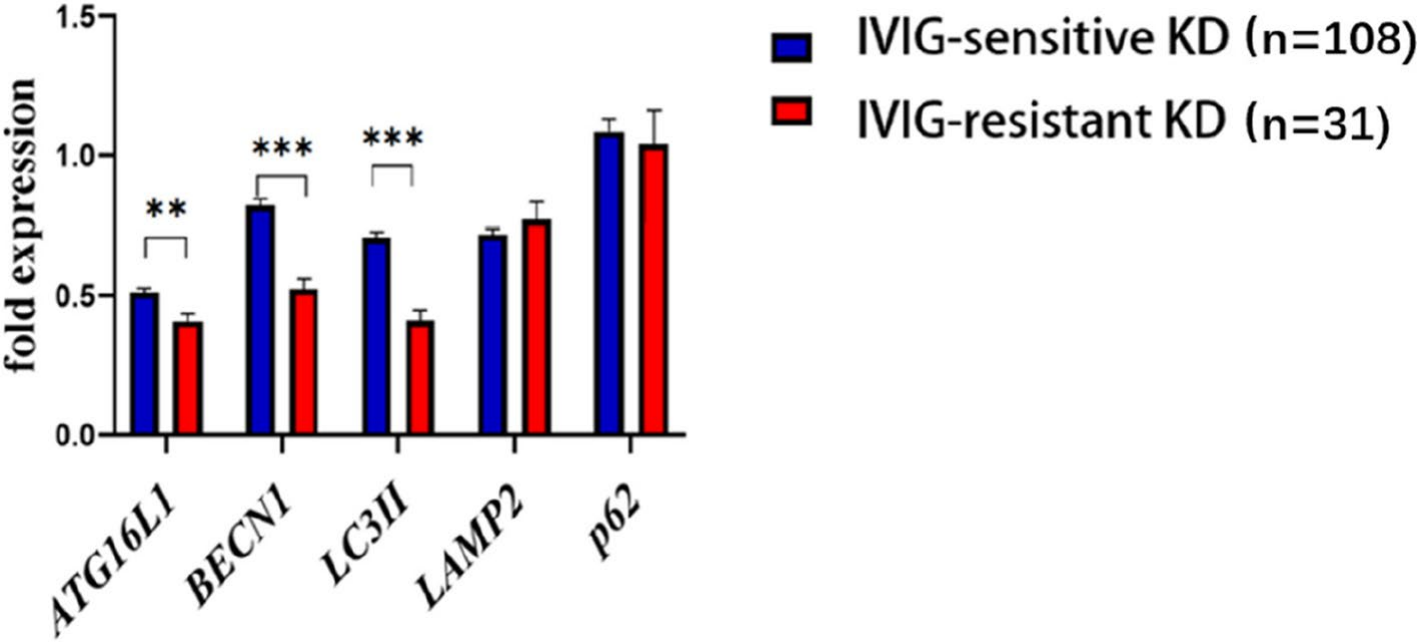
Analyses of mRNA autophagy markers in the peripheral white blood cells of Kawasaki disease (KD) patients

ATG16L1 0.42 (0.34, 0.50) vs. 0.49 (0.41,0.61), BECN1 0.46 (0.41, 0.63) vs. 0.83 (0.66,1), LC3II 0.41 ± 0.21 vs. 0.71 ± 0.19 were significantly downregulated in the IVIG-resistant Kawasaki disease group(*P* < 0.05), and *LAMP2*, *p62* were not significantly different (Table 5, Fig. 2).

**Table 5:** Autophagy indicators between IVIG-sensitive and IVIG-resistant KD children

Variable(s)	IVIG-sensitive (*n* = 108)	IVIG-resistant (*n* = 31)	Z	*P*
*ATG16L1*	0.49 (0.42,0.61)	0.41 (0.34,0.50)	3.095	0.002*
*BECN1*	0.83 (0.66,1)	0.46 (0.41,0.62)	5.772	< 0.001*
*LC3II*	0.71 ± 0.19	0.41 ± 0.21	6.930	< 0.001*
*LAMP2*	0.7 (0.58,0.81)	0.79 (0.52,1.03)	0.906	0.365
*p62*	1.09 ± 0.48	1.04 ± 0.68	0.433	0.666

^*^P < 0.05

### Correlation analysis of autophagic markers with clinical indices

*BECN1* was positively correlated with *ATG16L1*, while the incidence of IVIG-resistant Kawasaki disease, CAL, AST, NK% were negatively correlated with it (*p* < 0.05). RBC, HB, CK, CK-MB, ALB, A/G, LC3II, ATG16L1 were positively correlated with BECN1; the incidence of IVIG-resistant Kawasaki disease, length of stay, lymph node enlargement, perianal desquamation, CAL, IL-4, IL-6, CRP, PCT, SF, CD3 + CD4 + T, and r-GT were negatively correlated with BECN1. LC3II was positively correlated with BECN1 and negatively correlated with the incidence of IVIG-resistant Kawasaki disease, days in hospital, lymph node enlargement, IgG, and CAL (Tables 6 and 7).

**Table 6:** Correlation analysis of BECN1

Items	*BECN1*	
	*P*	r
Groups	0.000	-0.491
Length of hospitalization	0.002	-0.256
Enlarged lymph nodes	0.019	-0.2
Perianal peeling	0.008	-0.225
IL-4	0.018	-0.202
IL-6	0.007	-0.228
CRP	0.012	-0.212
RBC	0.003	0.249
HB	0.003	0.248
CK	0.013	0.21
CK-MB	0.039	0.175
CD3 + CD4 + T	0.031	-0.187
CAL	0.000	-0.343
ALB	0.004	0.241
A/G	0.031	0.183
r-GT	0.003	-0.247
PCT	0.013	-0.258
SF	0.015	-0.224
*LC3II*	0.006	0.231
*ATG16L1*	0.01	0.217

**Table 7:** Correlation analysis of LC3II

Items	*LC3II*	
	*P*	r
Groups	0.000	-0.500
Length of hospitalization	0.000	-0.364
Enlarged lymph nodes	0.018	-0.201
CAL	0.002	-0.262
IgG	0.046	-0.178
*BECN1*	0.006	0.231

### Independent risk factors for IVIG resistant KD

A one-way logistic regression analysis was performed on 16 indicators that differed between the IVIG-sensitive and IVIG-resistant groups, and significant correlations were found for 12 items: days in hospital, HB, RBC, ALB, GLB, A/G, CK, ESR, IgG, *ATG16L1*, *BECN1*, and *LC3II*. The 12 indicators associated with IVIG unresponsive Kawasaki disease were included in a multifactorial logistic regression analysis using forward stepwise regression, and the results showed that the expression of ESR, *BECN1*, and *LC3II* mRNA were independent risk factors for IVIG-resistant Kawasaki disease (Table 8).

**Table 8:** Single-factor logistic regression analysis

Items	B	SE	Wal	Df	*P*	Exp(B) (95%CI)
Length of hospitalization	0.346	0.092	14.215	1	0.000	1.41 (1.18,1.69)
HB	-0.54	0.018	9.471	1	0.002	0.95 (0.92,0.98)
RBC	-1.901	0.588	10.442	1	0.001	0.15 (0.05,0.47)
ALT	0.003	0.003	1.041	1	0.308	1 (1,1.01)
AST	0.006	0.004	2.881	1	0.090	1 (1.00,1.01)
ALB	-0.138	0.043	10.58	1	0.002	0.87 (0.80, 0.95)
GLB	0.096	0.028	12.037	1	0.001	1.10 (1.04, 1.16)
A/G	-1.118	0.527	12.788	1	0.000	0.15 (0.05,0.42)
r-GT	0.002	0.003	0.794	1	0.373	1.00 (1.00, 1.01)
CK	-0.015	0.007	5.045	1	0.025	0.99 (0.97,0.99)
ESR	0.02	0.007	8.058	1	0.005	1.02 (1.01,1.04)
PCT	0.034	0.035	0.938	1	0.333	1.03 (0.97, 1.11)
IgG	0.129	0.037	12.126	1	< 0.001	1.14 (1.06,1.22)
*ATG16L1*	-5.092	1.613	9.972	1	0.002	0.006 (0, 0.145)
*BECN1*	-5.655	1.17	23.346	1	< 0.001	0.004 (0,0.035)
*LC3II*	-7.262	1.427	25.894	1	0.001	0.001 (0,0.002)

### Predictive modeling

**Fig. 3:**
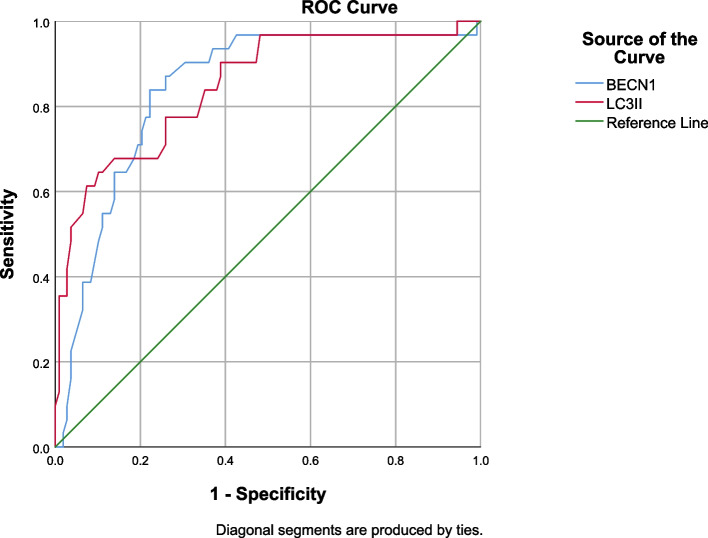
ROC curve for *LC3II* and *BECN1* between IVIG-resistant and IVIG-sensitive KD children

To ensure that the OR risk of each indicator was in the same direction, the transformed dichotomous indicators were assigned a reasonable value (0,1), and all the transformed indicators were brought into the logistic regression model again to obtain the OR of each dichotomous indicator. The OR of each dichotomous indicator was obtained by bringing all the transformed indicators into the logistic regression model, and the approximate score was assigned according to its size (Table 5). When the cut-off value of 0.645 was taken for *BECN1*, the sensitivity was 83.87%, the specificity was 77.78%, the Youden's index was 0.61, and the AUC was 0.841. When the cut-off value of 0.481 is taken for *LC3II*, the sensitivity is 67.74%, the specificity is 85.19%, the Youden index is 0.53, the AUC is 0.846 (Fig. 3).

**Fig. 4:**
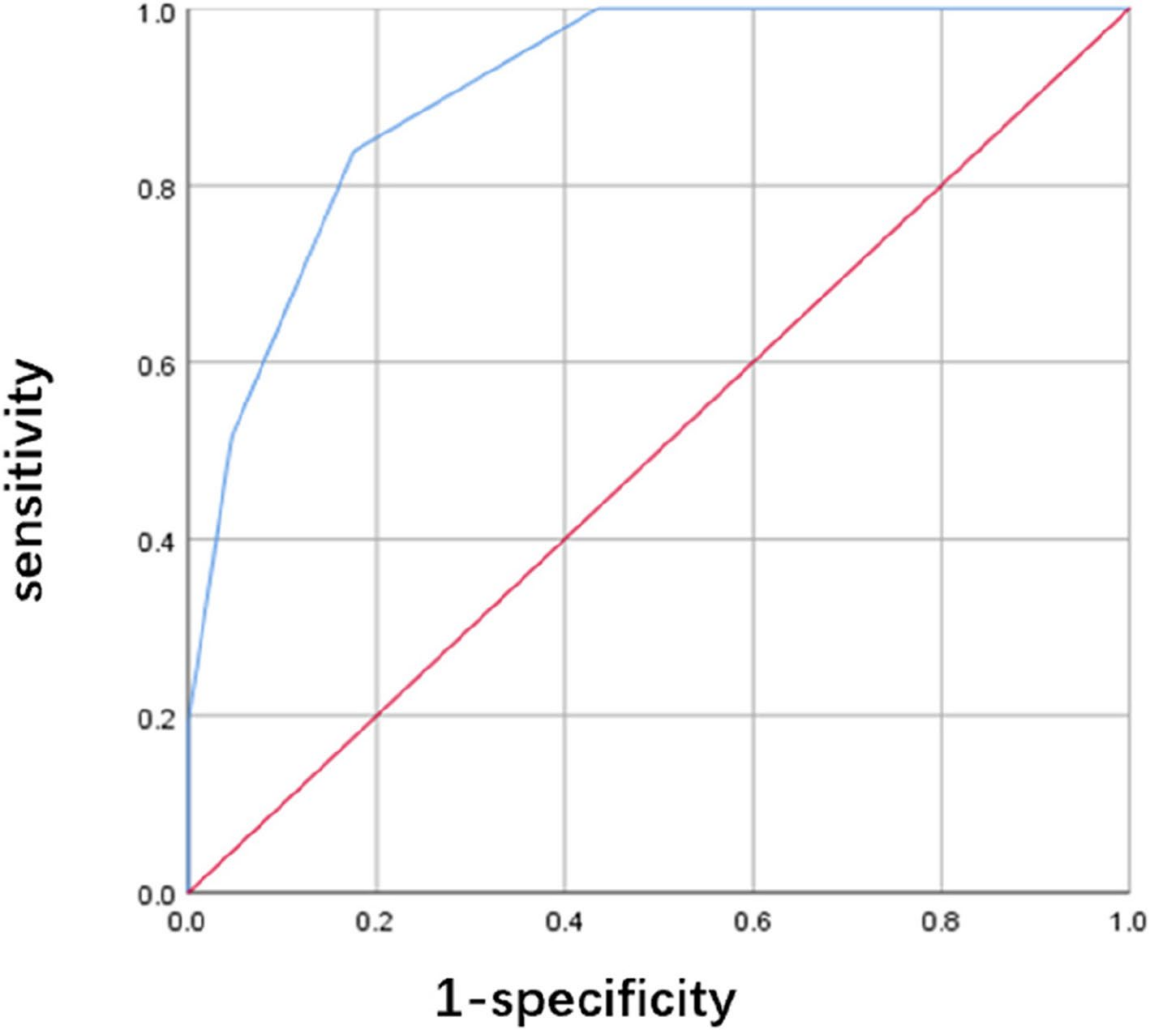
ROC curve for scoring model between IVIG-resistant and IVIG-sensitive KD children

A new scoring system was established based on the respective regression coefficients as follows: ESR ≥ 79.5 mm/h (1 point), *BECN1* ≤ 0.645 (1 point), *LC3II* ≤ 0.481 (2 points), 0–1 as low risk, ≥ 2 as high risk. Applied to this group of study subjects, sensitivity 87.10%, specificity 83.33%, positive predictive value 60.00%, negative predictive value 95.74%, Youden index 0.70, AUC 0.9 (Table 9, Fig. 4).

**Table 9:** Validation of the scoring model

Items	**Cutoff**	**Point**	**OR (95%CI)**	***P***
ESR	79.5	1 (> = 79.5), 0(< 79.5)	20.37 (3.737,111.044)	< 0.001
*BECN1*	0.645	1 (< = 0.645), 0(> 0.645)	15.766 (4.291,57.993)	< 0.001
*LC3II*	0.481	2 (< = 0.481), 0(> 0.481)	41.61 (7.764,223.006)	< 0.001

## Discussion

Our study showed that there was a significant reduction in the expression of *ATG16L1, BECN1,* and *LC3II* in the IVIG-resistant KD group compared with IVIG-sensitive KD group. And ESR, *BECN1*, and *LC3II* mRNA expression are independent risk factors for IVIG-resistant Kawasaki disease. We used these three indicators to establish a logistic regression model for predicting IVIG-resistant Kawasaki disease and assigned scores with ESR ≥ 79.5 mm/h (1 point), *BECN1* ≤ 0.645 (1 point), *LC3II* ≤ 0.481 (2 points), with 0–1 being low risk for IVIG-resistant and ≥ 2 being high risk. Applied to this group of study subjects, sensitivity 87.10%, specificity 83.33%, positive predictive value 60.00%, negative predictive value 95.74%, Youden index 0.70, AUC 0.9.

Autophagy is an indispensable biological process in mammals and plays a role in both pathological and physiological processes [20]. Autophagy degrades intracellular components by delivering them to lysosomes mainly through the assembly of autophagosomes in membrane-bound vesicle intermediates [21]. Autophagy plays a key role in pathogen invasion; on the one hand, it regulates antigen presentation to activate acquired immunity and enhance memory T-cell development [22]. On the other hand, it regulates cytokine signaling and promotes innate immunity [23]. There is growing evidence that autophagy is actively involved in endothelial dysfunction and vascular endothelial injury in pathophysiological processes of diseases such as atherosclerosis, sepsis and diabetes [24–26]. However, autophagy has shown conflicting results in cardiac diseases; on the one hand, it has been reported in the literature that autophagy promotes cardiomyocyte recovery and alleviates myocardial ischemia and reperfusion injury [27, 28], and on the other hand, autophagy increases cell death and exacerbates atherosclerosis [16]. The mRNA levels of autophagy markers (*LC3II, BECLIN1* and *ATG16L1*) were currently reported to be downregulated in leukocytes of children with KD in Taiwan, and significantly increased after immunoglobulin treatment [18].

*ATG16L1* interacts with the Atg12-Atg5 complex in the second step of autophagy and leads to the formation of Atg12-Atg5-Atg16 tetramers through its own zwitterionisation. These tetramers play a role in the extension of the autophagic precursor membrane. Therefore, early autophagosome formation can be detected by *ATG16L1*, and *ATG16L1* expression is upregulated when autophagy is enhanced [29]. It has been documented that in the leukocytes of CAL-injured children with Kawasaki disease, the expression of ATG16L1 continues to decrease even after treatment, suggesting that ATG16L1 may be involved in the process of CAL [18]. In our study, we found that the levels of *ATG16L1* were significantly downregulated in all of the KD groups, and there was a significant decrease in ATG16L1 expression in the IVIG- resistant KD group. But it is not the independent risk factors for IVIG-resistant Kawasaki disease.

*BECN1* regulates the signal transduction pathway at the initiation of autophagy. Normally, *BECN1* interacts with Bcl-2, when autophagy is inhibited. When cells are exposed to autophagy-inducing conditions, the pro-apoptotic molecule BH3 dissociates *BECN1* from Bcl-2. *BECN1* binds to PIK3C3/Vps34, which then activates autophagy [30], a process that plays an important role in the early stages of autophagosome formation [31]. A 2023 literature reported that macrophages inhibit autophagy through NET-mediated EGFR-BECN1 signaling to accelerate inflammatory vesicle activity thereby promoting atherosclerosis formation [32], and BECN1-dependent autophagy ameliorates lung injury and inflammation in sepsis and may protect the heart [33, 34]. In the present study, BECN1 expression was found to be reduced in Kawasaki disease, a result consistent with that reported by Huang [18]. Moreover, in our study it was significantly downregulated in IVIG-resistant Kawasaki disease compared with the IVIG-sensitive group, was an independent risk factor for IVIG-resistant Kawasaki disease, and was significantly correlated with CAL, suggesting that BECN1 may be related to the pathogenesis of IVIG-resistant Kawasaki disease and even the development of CAL. In a mouse sepsis model, it was found that blocking hepatic autophagy enhances impaired liver function and accelerates the time to death, and hepatic autophagy can play a protective role against organ failure [35], suggesting that autophagy may protect liver function, and the mechanism may be the same in IVIG-resistant Kawasaki disease. Inflammatory indicators such as IL-4, IL-6, CRP, PCT, and SF are negatively correlated with them, suggesting that a high inflammatory response may inhibit the activation of cellular autophagy. In recent years, it has been reported in the literature that IL-4 can inhibit the activation of autophagy, which is consistent with our study [14]. SF is a reactive protein positively associated with systemic inflammation, however, the clinical diagnostic significance in Kawasaki disease is still unclear. It has been shown that SF is significantly elevated in the acute phase of Kawasaki disease and further elevated when coronary injury occurs [36], and autophagy is inhibited in Kawasaki disease mouse models where coronary injury occurs, so autophagy may also be associated with elevated SF. In adult Still's disease, which also has a systemic inflammatory response, SF was detected to be significantly elevated, and autophagy can be enhanced by pharmacological treatment to reduce the inflammatory response [37], so it may be regulated by a similar signaling pathway in propionic sphere IVIG resistant KD.

*LC3II* binds to autophagic vesicles to form autophagosomes and is distributed on autophagic membranes, and is a biomarker of autophagosomes.*LC3II* content or *LC3II/LC3I* ratio is positively correlated with the number of autophagosomes and can be used to assess the level of autophagy in cells [38]*. LC3II* is involved in a variety of diseases, and has a certain protective role [39, 40]. In the acute phase of Kawasaki disease, the expression of peripheral blood *LC3II* was significantly reduced, which is consistent with our study. It has even been shown that resveratrol increases the expression of *ATG16L1* and *LC3II* by inducing autophagy, thus enhancing the anti-inflammatory effect of human coronary endothelial cells and even preventing the development of coronary aneurysms [41]. In the present study, compared with IVIG sensitive KD, *LC3II* expression was found to be significantly reduced in IVIG-resistant Kawasaki disease and was an independent risk factor for IVIG-resistant Kawasaki disease.

ESR is a classic marker of inflammation, and in a meta-analysis that included 4,442 cases, the ESR was significantly higher in the IVIG-resistant Kawasaki disease than in the IVIG-sensitive group [42]. By geographic region, ESR was significantly different in Chinese patients, while it was not in Japan and Korea [42]. It has been shown that serum in KD patients presents a redox reaction that disrupts RBC homeostasis, and its structural and functional alterations contribute to accelerated ESR, which leads to adverse events such as thrombosis and anemia, which is consistent with the decrease in RBC and HGB and increase in ESR in this study.

However, there are still some limitations of our study. Firstly, this was a single-center study and the sample size of enrolled patients was not large enough. Further multicenter studies are needed to validate the predictive efficiency in the future, and secondly, we used leukocytes from children rather than PBMC cells and the method used was qPCR, which may require additional laboratory methods for validation.

## Conclusion

The autophagy markers *ATG16L1*, *BECN1*, and *LC3II* are downregulated in the expression of IVIG-resistant Kawasaki disease. *ESR, BECN1,* and *LC3II* mRNAs are independent risk factors for IVIG-resistant Kawasaki disease and may be involved in the development of IVIG-resistant Kawasaki disease. This study established a new model that can be used to predict IVIG-resistant Kawasaki disease, and future validation in a larger population is needed.

## Data Availability

All of the material is owned by the authors and/or no permissions are required. They can contact the corresponding author.
